# Cardiovascular Safety of Anti-Sclerostin Therapy in Chronic Kidney Disease

**DOI:** 10.3390/metabo11110770

**Published:** 2021-11-10

**Authors:** Daniel Cejka

**Affiliations:** Department of Medicine III: Nephrology, Transplantation Medicine, Rheumatology, Geriatrics, Ordensklinikum Linz—Elisabethinen Hospital, Fadingerstraße 1, 4020 Linz, Austria; daniel.cejka@ordensklinikum.at; Tel.: +43-732-7676-4300; Fax: +43-732-7676-4306

**Keywords:** sclerostin, romosozumab, chronic kidney disease (CKD), chronic kidney disease–mineral and bone disorder (CKD–MBD), cardiovascular safety

## Abstract

The significance of sclerostin for bone and cardiovascular health in patients with chronic kidney disease (CKD) is complex and incompletely understood. Experimental evidence suggests that anti-sclerostin therapy shows diminished efficacy on bone in the setting of CKD. Limited clinical evidence suggests that the osteoanabolic and anti-resorptive activity is attenuated, but hypocalcemia is more prevalent in patients with advanced CKD (eGFR < 30 mL/min) treated with anti-sclerostin (romosozumab) therapy as compared to patients without kidney disease. Furthermore, sclerostin is prominently expressed in uremic arteries. Whether the inhibition of sclerostin has adverse effects on cardiovascular health in CKD is currently unknown. This review summarizes the current understanding of the physiology and pathophysiology of sclerostin in CKD, with a focus on the cardiovascular safety of anti-sclerostin therapy in patients with or without CKD.

## 1. Osteoporosis in CKD

Renal osteodystrophy (ROD) is a type of secondary osteoporosis which may occur at the early stages of chronic kidney disease (CKD), but typically occurs in patients with advanced CKD (Stage IV, eGFR < 30 mL/min). Patients with renal osteodystrophy may have alterations in bone volume, bone turnover or bone mineralization, which may occur individually or in different constellations. Hence, the turnover, mineralization, and volume-classification (TMV) of renal osteodystrophy was introduced in 2006, with the aim of the harmonization of the reporting of bone histology results for scientific research and clinical patient care [1]. Because ROD comes in different subtypes, with sometimes diametrically different characteristics (e.g., high or low bone turnover), it is not surprising that there is no “one-size-fits-all” treatment for ROD. For instance, the current notion is that high bone turnover should be treated with anti-resorptive agents, while low bone turnover should be treated with turnover-activating osteoanabolic therapies.

## 2. Sclerostin as Marker of Bone Turnover in CKD

Bone biopsy is the gold standard for the precise diagnosis of ROD. Because of all the well-known limitations of bone biopsies, non-invasive methods for the diagnosis of ROD have been intensively studied over the past decades. Numerous markers of bone turnover, some of which are nowadays available in clinical routine (such as bone-specific alkaline phosphatase, osteocalcin and carboxy-terminal collagen crosslinks) have been studied for the non-invasive diagnosis of ROD, with overall mixed results. (e.g., [2]). Parathyroid hormone (PTH) is a major determinant of bone turnover in advanced CKD. Because PTH signaling inhibits the expression of sclerostin in osteocytes and osteoblasts, serum sclerostin levels were studied as possible markers of bone turnover in CKD. Initially, as with all novel diagnostic parameters, sclerostin was measured with non-commercial assays. Consequently, a number of sclerostin assays have become commercially available. Assays by different manufacturers yield different results in terms of absolute sclerostin concentrations in serum. Therefore, studies using different sclerostin assays cannot be compared directly. However, commercially available sclerostin assays show good correlations between each other. Thus, sclerostin measurements with these commercial assays are probably valid (indeed measuring sclerostin) and inter-individual variability within a given cohort may be correlated with bone turnover [3,4,5]. However, bone biopsy studies in CKD patients showed only a moderate predictive value of sclerostin on bone turnover [6,7] or bone cellularity (as a proxy of bone turnover) [3], without added diagnostic value to the already established markers of bone turnover. Glucocorticoid treatment (commonly used in kidney transplantation) seems to attenuate the moderate association between sclerostin and bone turnover even further [8], rendering serum sclerostin measurements even less useful for the assessment of bone turnover.

## 3. Serum Sclerostin Is Increased in CKD

Serum sclerostin levels are markedly higher in CKD patients compared to healthy controls [9,10,11]. Two possible mechanisms were discussed to explain this finding: one being the increased expression of sclerostin in uremia, the other being the decreased renal elimination (renal retention) of sclerostin in patients with a low glomerular filtration rate (GFR). In patients with CKD, the renal elimination of sclerostin (fractional as well as absolute excretion) was found to increase with declining renal function [12], suggesting that increased sclerostin levels in CKD are the result of increased production. The increased expression of sclerostin in bone with decreasing renal function was also demonstrated in a cross-sectional bone biopsy study [13], which is also in line with experimental data [14]. Interestingly, this Brazilian study [13] found the highest expression of sclerostin in bone in early-stage CKD (stage 2), with subsequent a decline in expression in patients with more advanced CKD. However, sclerostin expression in bone in dialysis patients was still approximately twice as high as in kidney-healthy controls [13]. Serum sclerostin levels decline after kidney transplantation (corresponding to the improved renal function), but paradoxically the expression of sclerostin in bone increases [15]. Taken together, serum sclerostin levels in CKD patients cannot be fully explained by the expression of sclerostin in bone cells.

Very interesting results have been reported in preclinical [16] and clinical [17] studies of acute kidney injury (AKI). Increased serum sclerostin concentrations (approximately 3-fold increase compared to kidney-healthy controls) have been found in de novo (due to sepsis) or in acute-on-chronic kidney disease [17]. Therefore, even short-term uremia seems to lead to increased sclerostin expression. Immobilization occurring with severe illness, which has been described to increase sclerostin expression due to mechanical unloading (e.g., after stroke [18]), may be an alternative explanation for the elevated serum sclerostin levels in AKI.

## 4. Serum Sclerostin in CKD: Bone or Vessels?

Consequently, the source of extra-skeletal sclerostin expression has been a matter of intensive research. Vasculature, more specifically the arterial vascular wall, is suspected to be the source of extra-skeletal sclerostin. Vascular smooth muscle cells (VSMCs) exposed to pro-calcifying conditions in vitro show increased expression of sclerostin [19]. Additionally, the elevated expression of sclerostin in calcifying aortic tissue was found in an in vitro model of uremic vasculopathy [20]. Both AKI [16] and CKD [21] were demonstrated to lead to increased vascular (aortic) expression of sclerostin in vivo. Finally, some [22,23], but not all [24] studies in patients found that serum sclerostin levels are predominantly correlated with arterial sclerostin expression. Taken together, it appears that in CKD the majority of sclerostin circulating in serum comes from the vasculature.

CKD patients show extremely high rates of cardiovascular events and cardiovascular death [25]. Vascular calcification, which is at least partly an active process where vascular smooth muscle cells transdifferentiate to an osteoblast-like phenotype, is also widely prevalent and occurs prematurely in CKD patients. The finding that the expression of sclerostin, which is an inhibitor of osteoblast activity, is substantially increased in the vasculature in CKD is at least remarkable, if not troublesome. Currently, the pathophysiologic function of sclerostin in uremic arteries is unclear. If sclerostin was actively inhibiting vascular calcification in CKD, the inhibition of sclerostin would be detrimental by promoting further vascular calcification [26]. On the other hand, it could also by hypothesized that the vascular expression of sclerostin is a marker of uremic damage without much influence on the damage by itself [26]. In that case, the inhibition of sclerostin would probably remain without adverse cardiovascular consequences.

Recently published data suggest that uremic vessels can have adverse effects on bone [27]: When a piece of uremic aorta from a rat with CKD was transplanted into a kidney-healthy animal, the bone mineral density of the (otherwise healthy) recipient animal decreased, mainly through the impaired mineralization of bone, with marked increases in osteopontin and ANKH (progressive ankylosis protein homolog) [27], both inhibitors of mineralization. Additionally, the expression of sclerostin in bone increased in the recipient animals [27]. This study provided experimental evidence that the so-called bone–vascular axis in CKD can be bidirectional, and is not a “one-way street”.

## 5. Sclerostin and Cardiovascular Events

Sclerostin was identified to be the affected gene in sclerosteosis/van Buchem’s disease [28,29,30]. Sclerosteosis is a rare genetic disease, which has been mostly described in Afrikaans people (descendants of Dutch colonists) in South Africa. Mutations in the sclerostin gene or the associated promoter region lead to a lack of functionally active sclerostin, resulting in bone overgrowth. Patients with sclerosteosis suffer from the sequelae of this bone overgrowth, especially through the narrowing of neuroforamina with consecutive palsies such as facial nerve paralysis or deafness. Without adequate treatment (craniotomy), patients with sclerosteosis die in the third or fourth decade of their life because of increased intracranial pressure [31]. Cardiovascular disease has not been described to be more frequent in patients with sclerosteosis, but epidemiological studies in sclerosteosis patients are surely hampered by the low prevalence of the disease. In experimental studies in mice, rats or monkeys, where sclerostin was either completely absent by genetic knock-out or diminished in availability by antibody treatment, no cardiovascular pathology was reported [32,33,34,35,36]. However, the mouse strain used for the sclerostin gene-knock-out studies (C57/Bl6) is widely known to be resistant to cardiovascular disease, which is a limitation of the murine studies in terms of cardiovascular pathology.

Recently, two larger epidemiological studies on the association between sclerostin gene polymorphisms, which are associated with a low sclerostin expression phenotype (thus mimicking therapeutic intervention in sclerostin levels) and cardiovascular disease have been published. Because these studies yielded conflicting results, it seems worthwhile to take a closer look: The first study by Bovijn et al. [37] was predominantly based on data from a British biobank. Two sclerostin alleles (rs7209826, prevalence in biobank: 40%; rs188810925, prevalence in biobank: 8%), which were associated with diminished expression of sclerostin in tibial arteries and aorta (data from bone was not available) and increased bone mineral density (BMD) in the lumbar spine (difference in LS-BMD per allele: rs7209826, 0.008 g/cm^2^; rs188810925, 0.016 g/cm^2^), as well as decreased fracture incidence, were selected. This gene effect on LS-BMD was then “scaled” (i.e., multiplied) to the effect of an anti-sclerostin antibody (romosozumab) therapy for 12 months on LS-BMD. Subsequently, the cardiovascular risk associated with these two sclerostin alleles was also “scaled” (multiplied) with the same factor as for LS-BMD. Using this approach, Bovijn et al. [37] reported significant associations between sclerostin gene variants (scaled to the effect on LS-BMD of romosozumab to mimic anti-sclerostin therapy) with major adverse cardiovascular events (MACE). Odds ratios (OR) for MACE ranged from 1.10 to 1.18. Additionally, these sclerostin gene variants were also found to be associated with arterial hypertension (OR = 1.12) and diabetes mellitus (OR = 1.15).

The second study conducted by Holdsworth et al. [38] (which was funded by the manufacturer of romosozumab) did not find an association between sclerostin gene variants and cardiovascular disease. Holdsworth and colleagues [38] studied three sets of sclerostin alleles. For the first sclerostin allele set (rs9899889, rs1107748, rs66838809), which is associated with reduced expression of sclerostin and increased BMD, no association with cardiovascular disease was identified using the data of two population-based studies of cardiovascular risk (CARDIoGRAMplusC4D and MEGASTROKE) encompassing over 1 million participants. Furthermore, these sclerostin variants were not associated with arterial hypertension or diabetes mellitus. Holdsworth and colleagues [38] also studied the gene variants used by Bovijn et al. [37] (rs7209826 and rs188810925), but again did not find an association with cardiovascular disease in a multivariate analysis. Finally, Holdsworth et al. [38] analyzed a third sclerostin allele set (rs2741856 and rs7217502) as part of a sensitivity analysis, and again could not find an association with cardiovascular disease.

How can these discrepancies be explained?

The results reported by Bovijn et al. [37] may be subject to residual confounding, as neither arterial hypertension nor diabetes mellitus was observed in sclerostin gene knockout animals or preclinical and clinical studies with anti-sclerostin antibodies. Holdsworth and colleagues [38] discussed that Bovijn et al. [37] possibly underestimated the effect of the interdependency of the sclerostin alleles studied, and that insufficient correction for multiple testing was performed, both leading to the underestimation of *p*-values.

Taken together, an association between low sclerostin expression and cardiovascular disease appears epidemiologically possible, but seems unlikely based on the current understanding of the functions of sclerostin and experimental data.

## 6. Sclerostin and Patient Outcome in CKD

A number of studies investigated the association between serum sclerostin and pulse-wave velocity (PWV, a measure of vascular stiffness and surrogate parameter for cardiovascular disease), as well as patient mortality.

The majority of studies published reported a direct association between serum sclerostin levels and PWV [39,40,41,42,43]. However, some studies did not find an association between serum sclerostin and PWV [44,45] or an “arterial stiffness index”, which is measured at the fingertip [46].

Regarding the association between serum sclerostin and mortality in CKD patients, the general picture is quite unclear. Direct [47,48,49,50,51,52,53], indirect [54,55,56,57,58] or no [59,60,61,62] association between serum sclerostin and mortality in CKD have been reported. This might at least in part be due to the heterogenous nature of the CKD populations studied (CKD without renal replacement therapy, dialysis patients, kidney transplant recipients). However, even when looking only at a relatively well-defined patient population such as dialysis patients, the available data remain contradictory. Again, an important question is whether these associations are causal or not. Deducing from the (very differing) results from these studies and assuming causality, the inhibition of sclerostin might confer beneficial, detrimental or no effects at all in CKD patients. Ultimately, this question can only be answered in prospective, randomized, controlled, interventional trials.

## 7. Sclerostin, BMD and Fractures in CKD

The association between serum sclerostin levels and BMD is well studied. All [10,46,63,64,65] but one study (in pediatric CKD dialysis patients [66]) reported a direct association between serum sclerostin and BMD. Given the physiologic function of sclerostin as an inhibitor of osteoblast function, this finding is definitely counter-intuitive. However, a positive association between serum sclerostin levels and BMD has also been very consistently reported in kidney-healthy cohorts (e.g., [67]), so it is obviously not specific to CKD. So far, explanations brought forward for this phenomenon remain somewhat unsatisfactory. The most common explanation is that sclerostin production correlates with osteocyte number and hence total bone mass. The more bone, the higher the sclerostin levels. Whether this explanation indeed reflects the real situation remains to be determined. As high sclerostin levels have been associated with prevalent vertebral fractures [68] and progressive bone loss [69,70] in dialysis patients, the situation becomes even more complicated.

## 8. Anti-Sclerostin Therapy

Romosozumab is an anti-sclerostin antibody which was approved for clinical use by the U.S. Food and Drug Administration (FDA) as well as the European Medicines Agency (EMA) in 2019. The advent of romosozumab represents a radical improvement in the therapeutic armamentarium for the treatment of osteoporosis. No other drug induces such large increases in BMD in all parts of the skeleton in such a short time (1 year of treatment). Romosozumab/anti-sclerostin antibody therapy shows both anti-resorptive and osteoanabolic properties, distinguishing it from other currently used osteoporosis drugs. So far, romosozumab has shown a very favorable safety profile. However, in patients with advanced CKD, this might be different.

## 9. Romosozumab and Cardiovascular Events

The efficacy and safety of romosozumab was studied in three large, international, randomized controlled trials. Two trials were conducted with postmenopausal women (acronyms: FRAME [71], ARCH [72]), and one in men with osteoporosis (acronym: BRIDGE [73]). In the largest study with romosozumab (FRAME, *n* = 7180 women) [71], no difference in cardiovascular events was found (romosozumab vs. placebo, *n* = 44 (1.2%) vs. *n* = 41 (1.1%)). However, when romosozumab was compared with alendronate (ARCH, *n* = 4093 women) [72], a slightly higher cardiovascular event rate was observed with romosozumab (*n* = 50, 2.5%) compared to alendronate (*n* = 38, 1.9%). In the BRIDGE study, where 245 men were randomized in a 2:1 fashion to receive romosozumab or placebo, eight cardiovascular events (4.9%) were noted in the romosozumab-group, while two cardiovascular events (2.5%) were observed in the placebo group. Meta-analysis of romosozumab trials regarding cardiovascular safety yielded conflicting results, which may be due to differences in the statistical methods applied (e.g., how to deal statistically with studies without an event) [74,75].

Because alendronate was the comparator to romosozumab in the ARCH study [72], a possible cardiovascular benefit of alendronate was proposed [76,77]. Indeed, beneficial effects of bisphosphonate therapy on the cardiovascular system have been suspected for quite some time, based on results from clinical registries (e.g., [78]). However, this proposed cardiovascular benefit of bisphosphonate treatment could not be substantiated in respective meta-analyses [79,80,81].

**Figure 1:**
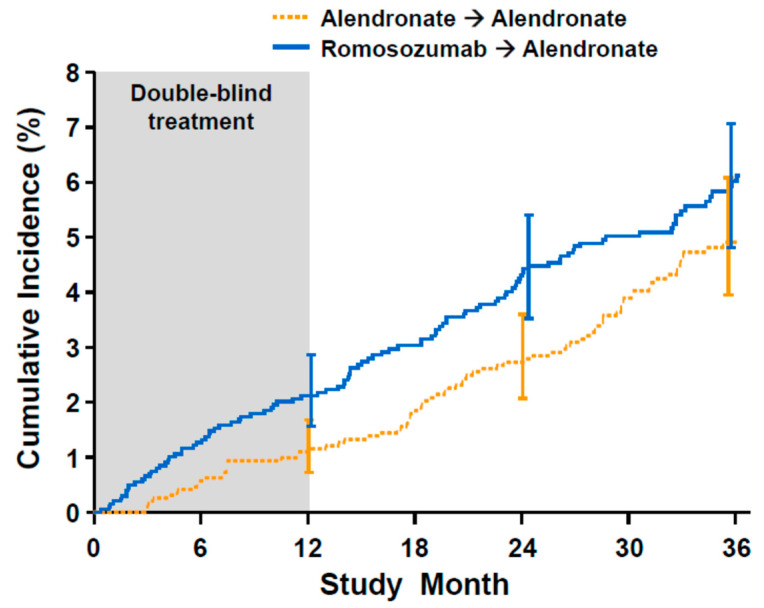
Time to first cardiovascular event in ARCH (romosozumab vs. alendronate), according to study arm. Note the absence of cardiovascular events in the first three months in the alendronate arm (source: FDA website [83], free for reproduction).

A close look at the cardiovascular event rate reported in ARCH (Figure 1) reveals interesting further insights: strikingly, there was no recorded cardiovascular event in the alendronate study arm. Such an immediate and potent effect on cardiovascular events by alendronate is neither mechanistically plausible nor supported by previous clinical observations. In the extension period after 1 year, where both study arms received alendronate, the cardiovascular event rates seem to run in parallel, without apparent changes in the cardiovascular event rate in patients switched from romosozumab to alendronate. Subsequently (study month 24–36), the cardiovascular event rate in the original alendronate arm seemed to increase. Thus, the differences in cardiovascular events between romosozumab and alendronate may be a result of chance.

To approach this question from a pathophysiological perspective, the expression of sclerostin in atherosclerotic plaques was studied in human vascular specimens in detail [38]. Of note, this study was again financed by the company marketing romosozumab. In this histological study, sclerostin expression was not located in or close to regions of atherosclerotic plaques, which are widely viewed as being crucial for plaque stability (fibrous cap, endothelium). Therefore, a mechanistic link between sclerostin or the inhibition of sclerostin and atherosclerotic plaque rupture seems unlikely.

Nevertheless, both FDA and EMA required post-marketing surveillance for romosozumab to further assess cardiovascular safety. The first results from post-marketing data have already been published [82], suggesting an increased cardiovascular risk for patients treated with romosozumab. However, romosozumab-treated patients were older and were more likely to take concomitant cardiovascular medication (anticoagulants, anti-platelet agents, antihypertensives) and therefore were probably more likely to have cardiovascular events compared to the control group. Until further data become available, romosozumab is contraindicated in patients with a history of myocardial infarction or stroke (labeling Evenity^®^, Breda, the Netherlands accessed on 9 December 2019).

## 10. Anti-Sclerostin Therapy in CKD

The effects of the inhibition of sclerostin on bone in CKD were assessed in several experimental studies. In mice with advanced CKD, no relevant differences in bone changes in response to uremia were observed between sclerostin knock-out animals and wild-type controls [36,84]. In both studies, no attempt was made to lower PTH levels (due to secondary hyperparathyroidism—sHPTH). In another study using CKD rats [85], treatment with an anti-sclerostin antibody again failed to have substantial effects on bone in animals with sHPTH. However, when sHPTH was suppressed using calcium supplementation, anti-sclerostin antibody therapy led to increases in bone volume [85].

Based on these studies, one might hypothesize that anti-sclerostin therapy in patients with advanced CKD (and sHPTH) would need to be combined with PTH-lowering treatment for efficacy. Whether treatment with active vitamin D (e.g., calcitriol, paricalcitol, alphacalcidol) or calcimimetics (cinacalcet, etelcalcetide) would be the better combination with romosozumab remains to be studied.

In the large studies investigating the efficacy and safety of romosozumab (FRAME, ARCH), some restrictions applied for the participation of patients with CKD. In FRAME [71], CKD was not an exclusion criterion, but PTH above the upper limit of normal was. As sHPTH occurs frequently in advanced CKD, this restriction probably led to the indirect exclusion of patients with advanced CKD. In ARCH [72], not only was PTH above the upper limit of normal an exclusion criterion, but patients with an eGFR < 35 mmL/min (MDRD formula) were also explicitly excluded from participation. As occurs so often, patients with advanced stage kidney disease (roughly CKD 4 and higher) were excluded from these studies, which is regrettable from the nephrologist’s point of view. However, keeping in mind the complex and unclear relationship between sclerostin and cardiovascular health in CKD, this may have been a good decision.

Based on the available data from the FRAME and ARCH studies, a post hoc analysis regarding the efficacy and safety of romosozumab in mild to moderate CKD was performed, but is available only as an abstract so far [86]. Patients were grouped according to their eGFR in normal renal function (eGFR > 90 mL/min), mild (eGFR 60–89 mL/min, equivalent to CKD stage 2) or moderate (eGFR 30–59 mL/min, equivalent to CKD stage 3) renal impairment. There was a trend of decreased efficacy of romosozumab (lower increase in BMD) with lower eGFR. In terms of cardiovascular safety, no association with renal function was found.

**Figure 2:**
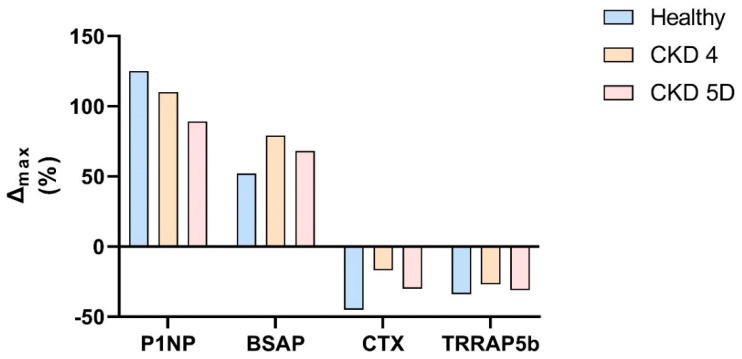
Peak percentual change in bone turnover markers from baseline after a single injection of romosozumab 210 mg, according to renal function. Adapted from [88]. Healthy—healthy controls; CKD 4—chronic kidney disease stage 4; CKD 5D—chronic kidney disease stage 5D (dialysis); P1NP—procollagen type 1 N-terminal propeptide; BSAP—bone-specific alkaline phosphatase; CTX—carboxy-terminal collagen crosslinks; TRAP5b—tartrate-resistant acid phosphatase 5b.

Regarding efficacy and safety in advanced CKD (eGFR < 30 mL/min, corresponding to CKD stage 4 or 5), information is scarce. Data from a phase I study have been published, but are available only in a clinical trial registry (clinicaltrials.gov) and the clinical trial registry of the manufacturer (amgentrials.com, accessed on 25 June 2021) [87,88]. This phase I study included 24 patients, divided into three groups (eight persons each) with normal renal function, CKD stage 4 or CKD on dialysis (CKD 5D). All participants received a single injection of 210 mg romosozumab, which is the currently approved dose. Follow-up was 85 days. Remarkably, all three patient groups showed increases in osteoblast markers and decreases in osteoclast markers. However, percent changes in bone turnover markers seemed to be somewhat diminished in CKD patients compared to healthy individuals (Figure 2). Data for BMD were available in only two participants and were not reported, which would probably be of very little use anyway given the low patient number and the short follow-up of less than 3 months.

**Figure 3:**
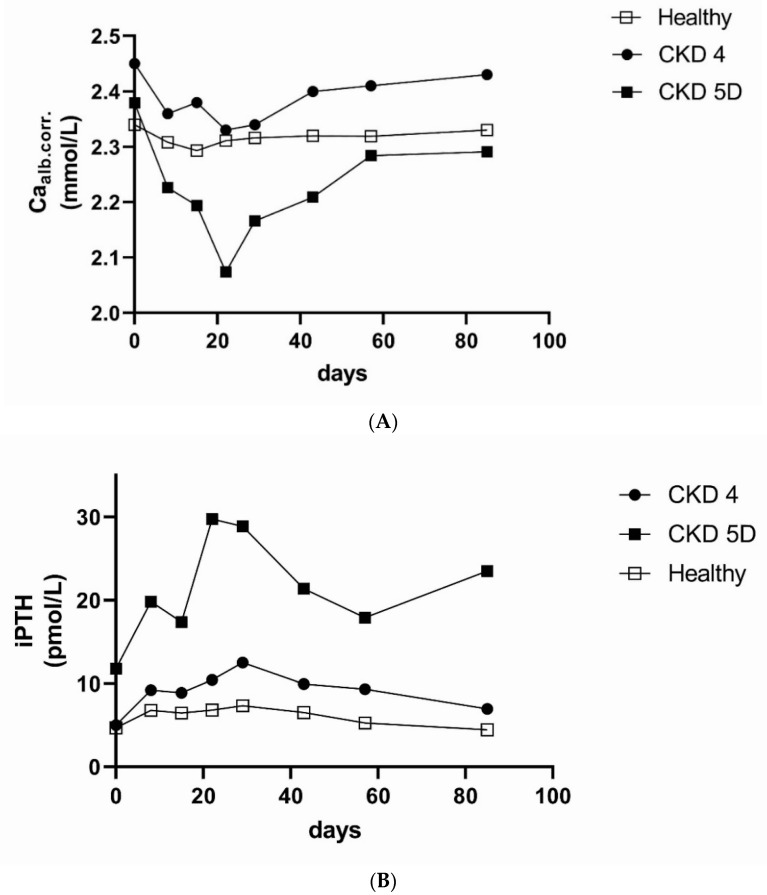
Mean values of (**A**) albumin-corrected calcium (Ca alb.corr.) and (**B**) intact parathyroid hormone (iPTH, log-scale) after a single injection of romosozumab 210 mg according to renal function. Adapted from [87]. Healthy—healthy controls; CKD 4—chronic kidney disease stage 4; CKD 5D—chronic kidney disease stage 5D (dialysis).

In terms of safety (Figure 3), decreases in albumin-corrected calcium were observed in CKD 4 patients and were even more pronounced in CKD 5D patients, with a nadir around 3 weeks after injection and a rebound after 1 (CKD 4) to 2 (CKD 5D) months to baseline values. In CKD 5D, the mean nadir of albumin-corrected calcium was 2.07 mmol/L, with 1 patient experiencing a grade 3 -hypocalcemia (1.5–1.75 mmol/L). In parallel to the development of hypocalcemia, there was a prominent (and physiologically expected) but transient increase in PTH. Interestingly, kidney-healthy individuals also experienced a marked increase in PTH (+94%) from the baseline. The pathophysiology behind the increased rate of hypocalcemia in CKD patients treated with romosozumab is currently unclear. It appears that the homeostasis of serum calcium is more dependent on bone turnover in CKD patients compared to kidney-healthy people. Because romosozumab has a dual mode of action (both osteoanabolic and antiresorptive) [89], increased uptake of calcium into newly formed bone as well as decreased calcium efflux from bone may result in decreased serum calcium levels. However, increased rates of hypocalcemia have been observed in CKD patients treated with denosumab [90], which is an anti-resorptive agent. Therefore, the anti-resorptive effect of romosozumab might play a dominant role in romosozumab-induced hypocalcemia in CKD patients.

Very recently, a Japanese group published a cohort study of 96 hemodialysis patients at high risk for fracture who were treated with romosozumab for 1 year [91]. Despite pre-treatment with bisphosphonates in the majority of patients, significant increases in LS-BMD (15.3 ± 12.9%) as well as FN-BMD (7.2 ± 8.3%) were observed in patients treated with romosozumab, whereas a cohort not treated with romosozumab (*n* = 55) showed no changes in LS-BMD or FN-BMD. Hypocalcemia was common in romosozumab-treated hemodialysis patients (9.5 ± 0.8 mg/dL at baseline to a nadir of 8.9 ± 0.7 mg/dL at 6 weeks after initiation), but was reversible and could be managed by increasing treatment with active vitamin D. Of note, the baseline value of iPTH was 152.3 ± 172.0 pg/mL, suggesting a very well-controlled secondary HTPH, which may have helped to mitigate the hypocalcemic effect of romosozumab treatment in hemodialysis patients. As compared to the control cohort, no increase in cardiovascular events was observed in romosozumab-treated patients.

## 11. Conclusions

For kidney-healthy patients, romosozumab is a very effective novel therapeutic option for the treatment of osteoporosis. So far, the cardiovascular safety profile is favorable, pending further reports from post-marketing surveillance. For patients with advanced CKD (stage 4/5), only very limited data regarding safety are available, while for effects on BMD there are no data available at all. Based on pathophysiologic considerations, the risk to benefit ratio of romosozumab may be less favorable in advanced stages of CKD. Therefore, romosozumab treatment in patients with advanced CKD (CKD stage 4 or 5, corresponding to an eGFR < 30 mL/min) should be performed only in the well-controlled setting of clinical studies, until further data become available.

## References

[1] Moe S., Drüeke T., Cunningham J., Goodman W., Martin K., Olgaard K., Ott S., Sprague S., Lameire N., Eknoyan G. (2006). Definition, Evaluation, and Classification of Renal Osteodystrophy: A Position Statement from Kidney Disease: Improving Global Outcomes (Kdigo). Kidney Int..

[2] Sprague S.M., Bellorin-Font E., Jorgetti V., Carvalho A.B., Malluche H.H., Ferreira A., D’Haese P.C., Drueke T.B., Du H., Manley T. (2015). Diagnostic Accuracy of Bone Turnover Markers and Bone Histology in Patients with Ckd Treated by Dialysis. Am. J. Kidney Dis..

[3] De Maré A., Verhulst A., Cavalier E., Delanaye P., Behets G.J., Meijers B., Kuypers D., D’Haese P.C., Evenepoel P. (2019). Clinical Inference of Serum and Bone Sclerostin Levels in Patients with End-Stage Kidney Disease. J. Clin. Med..

[4] Mause S.F., Deck A., Hennies M., Kaesler N., Evenepoel P., Boisvert W.A., Janssen U., Brandenburg V.M. (2016). Validation of Commercially Available Elisas for the Detection of Circulating Sclerostin in Hemodialysis Patients. Discoveries.

[5] Moyses M.R., Jamal S.A., Graciolli F.G., dos Reis L.M., Elias R.M. (2015). Can We Compare Serum Sclerostin Results Obtained with Different Assays in Hemodialysis Patients?. Int. Urol. Nephrol..

[6] Cejka D., Herberth J., Branscum A.J., Fardo D.W., Monier-Faugere M.C., Diarra D., Haas M., Malluche H.H. (2011). Sclerostin and Dickkopf-1 in Renal Osteodystrophy. Clin. J. Am. Soc. Nephrol..

[7] De Oliveira R.A., Barreto F.C., Mendes M., dos Reis L.M., Castro J.H., Britto Z.M., Marques I.D., Carvalho A.B., Moyses R.M., Jorgetti V. (2015). Peritoneal Dialysis Per Se Is a Risk Factor for Sclerostin-Associated Adynamic Bone Disease. Kidney Int..

[8] Boltenstal H., Qureshi A.R., Behets G.J., Lindholm B., Stenvinkel P., D’Haese P.C., Haarhaus M. (2019). Association of Serum Sclerostin with Bone Sclerostin in Chronic Kidney Disease Is Lost in Glucocorticoid Treated Patients. Calcif. Tissue Int..

[9] Pelletier S., Dubourg L., Carlier M.C., Hadj-Aissa A., Fouque D. (2013). The Relation between Renal Function and Serum Sclerostin in Adult Patients with Ckd. Clin. J. Am. Soc. Nephrol..

[10] Cejka D., Jager-Lansky A., Kieweg H., Weber M., Bieglmayer C., Haider D.G., Diarra D., Patsch J., Kainberger F., Bohle B. (2011). Sclerostin Serum Levels Correlate Positively with Bone Mineral Density and Microarchitecture in Haemodialysis Patients. Nephrol. Dial. Transplant..

[11] Lima F., Mawad H., El-Husseini A.A., Davenport D.L., Malluche H.H. (2019). Serum Bone Markers in Rod Patients across the Spectrum of Decreases in Gfr: Activin a Increases before All Other Markers. Clin. Nephrol..

[12] Cejka D., Marculescu R., Kozakowski N., Plischke M., Reiter T., Gessl A., Haas M. (2013). Renal Elimination of Sclerostin Increases with Declining Kidney Function. J. Clin. Endocrinol. Metab..

[13] Graciolli F.G., Neves K.R., Barreto F., Barreto D.V., Dos Reis L.M., Canziani M.E., Sabbagh Y., Carvalho A.B., Jorgetti V., Elias R.M. (2017). The Complexity of Chronic Kidney Disease-Mineral and Bone Disorder across Stages of Chronic Kidney Disease. Kidney Int..

[14] Sabbagh Y., Graciolli F.G., O’Brien S., Tang W., dos Reis L.M., Ryan S., Phillips L., Boulanger J., Song W., Bracken C. (2012). Repression of Osteocyte Wnt/Beta-Catenin Signaling Is an Early Event in the Progression of Renal Osteodystrophy. J. Bone Miner. Res..

[15] Araújo M.J.C.L.N., Marques I.D.B., Graciolli F.G., Fukuhara L., dos Reis L.M., Custódio M., Jorgetti V., Elias R.M., David-Neto E., Moysés R.M. (2019). Comparison of Serum Levels with Bone Content and Gene Expression Indicate a Contradictory Effect of Kidney Transplantation on Sclerostin. Kidney Int..

[16] Nordholm A., Mace M.L., Gravesen E., Hofman-Bang J., Morevati M., Olgaard K., Lewin E. (2018). Klotho and Activin a in Kidney Injury: Plasma Klotho Is Maintained in Unilateral Obstruction Despite No Upregulation of Klotho Biosynthesis in the Contralateral Kidney. Am. J. Physiol. Renal Physiol..

[17] Fayed A., Abdulazim D.O., Amin M., Elhadidy S., Samir H.H., Salem M.M., ElAzim I.M.A., el Hawary K.E.S., el Din U.A.S., Group Vascular Calcification (2021). Serum Sclerostin in Acute Kidney Injury Patients. Nefrologia.

[18] Gaudio A., Pennisi P., Bratengeier C., Torrisi V., Lindner B., Mangiafico R.A., Pulvirenti I., Hawa G., Tringali G., Fiore C.E. (2010). Increased Sclerostin Serum Levels Associated with Bone Formation and Resorption Markers in Patients with Immobilization-Induced Bone Loss. J. Clin. Endocrinol. Metab..

[19] Zhu D., Mackenzie N.C., Millan J.L., Farquharson C., MacRae V.E. (2011). The Appearance and Modulation of Osteocyte Marker Expression During Calcification of Vascular Smooth Muscle Cells. PLoS ONE.

[20] Bisson S.K., Ung R.V., Picard S., Valade D., Agharazii M., Lariviere R., Mac-Way F. (2019). High Calcium, Phosphate and Calcitriol Supplementation Leads to an Osteocyte-Like Phenotype in Calcified Vessels and Bone Mineralisation Defect in Uremic Rats. J. Bone Miner. Metab..

[21] Rukov J.L., Gravesen E., Mace M.L., Hofman-Bang J., Vinther J., Andersen C.B., Lewin E., Olgaard K. (2016). Effect of Chronic Uremia on the Transcriptional Profile of the Calcified Aorta Analyzed by Rna Sequencing. Am. J. Physiol. Renal Physiol..

[22] Zhou H., Yang M., Li M., Cui L. (2017). Radial Artery Sclerostin Expression in Chronic Kidney Disease Stage 5 Predialysis Patients: A Cross-Sectional Observational Study. Int. Urol. Nephrol..

[23] Li M., Zhou H., Yang M., Xing C. (2019). Relationship between Serum Sclerostin, Vascular Sclerostin Expression and Vascular Calcification Assessed by Different Methods in Esrd Patients Eligible for Renal Transplantation: A Cross-Sectional Study. Int. Urol. Nephrol..

[24] Qureshi A.R., Olauson H., Witasp A., Haarhaus M., Brandenburg V., Wernerson A., Lindholm B., Soderberg M., Wennberg L., Nordfors L. (2015). Increased Circulating Sclerostin Levels in End-Stage Renal Disease Predict Biopsy-Verified Vascular Medial Calcification and Coronary Artery Calcification. Kidney Int..

[25] Go A.S., Chertow G.M., Fan D., McCulloch C.E., Hsu C.y. (2004). Chronic Kidney Disease and the Risks of Death, Cardiovascular Events, and Hospitalization. N. Engl. J. Med..

[26] Brandenburg V.M., Verhulst A., Babler A., D’Haese P.C., Evenepoel P., Kaesler N. (2019). Sclerostin in Chronic Kidney Disease-Mineral Bone Disorder Think First before You Block It!. Nephrol. Dial. Transplant..

[27] Mace M.L., Gravesen E., Nordholm A., Egstrand S., Morevati M., Nielsen C., Kjaer A., Behets G., D’Haese P., Olgaard K. (2021). Chronic Kidney Disease-Induced Vascular Calcification Impairs Bone Metabolism. J. Bone Miner. Res..

[28] Brunkow M.E., Gardner J.C., Van Ness J., Paeper B.W., Kovacevich B.R., Proll S., Skonier J.E., Zhao L., Sabo P.J., Fu Y. (2001). Bone Dysplasia Sclerosteosis Results from Loss of the Sost Gene Product, a Novel Cystine Knot-Containing Protein. Am. J. Hum. Genet..

[29] Balemans W., Ebeling M., Patel N., Van H.E., Olson P., Dioszegi M., Lacza C., Wuyts W., van Den E.J., Willems P. (2001). Increased Bone Density in Sclerosteosis Is Due to the Deficiency of a Novel Secreted Protein (Sost). Hum. Mol. Genet..

[30] Balemans W., Patel N., Ebeling M., Van Hul E., Wuyts W., Lacza C., Dioszegi M., Dikkers F.G., Hildering P., Willems P.J. (2002). Identification of a 52 Kb Deletion Downstream of the Sost Gene in Patients with Van Buchem Disease. J. Med. Genet..

[31] Hamersma H., Gardner J., Beighton P. (2003). The Natural History of Sclerosteosis. Clin. Genet..

[32] Li X., Ominsky M.S., Warmington K.S., Morony S., Gong J., Cao J., Gao Y., Shalhoub V., Tipton B., Haldankar R. (2009). Sclerostin Antibody Treatment Increases Bone Formation, Bone Mass, and Bone Strength in a Rat Model of Postmenopausal Osteoporosis*. J. Bone Miner. Res..

[33] Ominsky M.S., Vlasseros F., Jolette J., Smith S.Y., Stouch B., Doellgast G., Gong J., Gao Y., Cao J., Graham K. (2010). Two Doses of Sclerostin Antibody in Cynomolgus Monkeys Increases Bone Formation, Bone Mineral Density, and Bone Strength. J. Bone Miner. Res..

[34] Li X., Ominsky M.S., Niu Q.T., Sun N., Daugherty B., D’Agostin D., Kurahara C., Gao Y., Cao J., Gong J. (2008). Targeted Deletion of the Sclerostin Gene in Mice Results in Increased Bone Formation and Bone Strength. J. Bone Miner. Res..

[35] Turk J.R., Deaton A.M., Yin J., Stolina M., Felx M., Boyd G., Bienvenu J.G., Varela A., Guillot M., Holdsworth G. (2020). Nonclinical Cardiovascular Safety Evaluation of Romosozumab, an Inhibitor of Sclerostin for the Treatment of Osteoporosis in Postmenopausal Women at High Risk of Fracture. Regul. Toxicol. Pharmacol..

[36] Kaesler N., Verhulst A., de Mare A., Deck A., Behets G.J., Hyusein A., Evenepoel P., Floege J., Marx N., Babler A. (2018). Sclerostin Deficiency Modifies the Development of Ckd-Mbd in Mice. Bone.

[37] Bovijn J., Krebs K., Chen C.Y., Boxall R., Censin J.C., Ferreira T., Pulit S.L., Glastonbury C.A., Laber S., Millwood I.Y. (2020). Evaluating the Cardiovascular Safety of Sclerostin Inhibition Using Evidence from Meta-Analysis of Clinical Trials and Human Genetics. Sci. Transl. Med..

[38] Holdsworth G., Staley J.R., Hall P., van Koeverden I., Vangjeli C., Okoye R., Boyce R.W., Turk J.R., Armstrong M., Wolfreys A. (2021). Sclerostin Downregulation Globally by Naturally Occurring Genetic Variants, or Locally in Atherosclerotic Plaques, Does Not Associate with Cardiovascular Events in Humans. J. Bone Miner. Res..

[39] Desjardins L., Liabeuf S., Oliveira R.B., Louvet L., Kamel S., Lemke H.D., Vanholder R., Choukroun G., Massy Z.A., Group European Uremic Toxin Work (2014). Uremic Toxicity and Sclerostin in Chronic Kidney Disease Patients. Nephrol. Ther..

[40] Hsu B.G., Liou H.H., Lee C.J., Chen Y.C., Ho G.J., Lee M.C. (2016). Serum Sclerostin as an Independent Marker of Peripheral Arterial Stiffness in Renal Transplantation Recipients: A Cross-Sectional Study. Medicine.

[41] Jin S., Zhu M., Yan J., Fang Y., Lu R., Zhang W., Zhang Q., Lu J., Qi C., Shao X. (2016). Serum Sclerostin Level Might Be a Potential Biomarker for Arterial Stiffness in Prevalent Hemodialysis Patients. Biomark. Med..

[42] Stavrinou E., Sarafidis P.A., Koumaras C., Loutradis C., Giamalis P., Tziomalos K., Karagiannis A., Papagianni A. (2019). Increased Sclerostin, but Not Dickkopf-1 Protein, Is Associated with Elevated Pulse Wave Velocity in Hemodialysis Subjects. Kidney Blood Press. Res..

[43] Wu C.F., Hou J.S., Wang C.H., Lin Y.L., Lai Y.H., Kuo C.H., Liou H.H., Tsai J.P., Hsu B.G. (2020). Serum Sclerostin but Not Dkk-1 Correlated with Central Arterial Stiffness in End Stage Renal Disease Patients. Int. J. Environ. Res. Public Health.

[44] Gelir G.K., Sengul S., Nergizoglu G., Erturk S., Duman N., Kutlay S. (2018). Is Sclerostin Level Associated with Cardiovascular Diseases in Hemodialysis Patients?. Blood Purif..

[45] Petrovic M., Baralic M., Brkovic V., Arsenovic A., Stojanov V., Lalic N., Stanisavljevic D., Jankovic A., Radivojevic N., Pejanovic S. (2020). Significance of Acpwv for Survival of Hemodialysis Patients. Medicina.

[46] Thambiah S., Roplekar R., Manghat P., Fogelman I., Fraser W.D., Goldsmith D., Hampson G. (2012). Circulating Sclerostin and Dickkopf-1 (Dkk1) in Predialysis Chronic Kidney Disease (Ckd): Relationship with Bone Density and Arterial Stiffness. Calcif. Tissue Int..

[47] Chen A., Sun Y., Cui J., Zhao B., Wang H., Chen X., Mao Y. (2018). Associations of Sclerostin with Carotid Artery Atherosclerosis and All-Cause Mortality in Chinese Patients Undergoing Maintenance Hemodialysis. BMC Nephrol..

[48] Kalousova M., Dusilova-Sulkova S., Kubena A.A., Zakiyanov O., Tesar V., Zima T. (2019). Sclerostin Levels Predict Cardiovascular Mortality in Long-Term Hemodialysis Patients: A Prospective Observational Cohort Study. Physiol. Res..

[49] Zou Y., Yang M., Wang J., Cui L., Jiang Z., Ding J., Li M., Zhou H. (2020). Association of Sclerostin with Cardiovascular Events and Mortality in Dialysis Patients. Ren. Fail..

[50] Zeng S., Slowinski T., Pommer W., Hasan A.A., Gaballa M.M.S., Lu Y., Kramer B.K., Hocher B. (2020). Sclerostin Is an Independent Risk Factor for All-Cause Mortality in Kidney Transplant Recipients. Clin. Exp. Nephrol..

[51] Stavrinou E., Sarafidis P.A., Loutradis C., Memmos E., Faitatzidou D., Giamalis P., Koumaras C., Karagiannis A., Papagianni A. (2021). Associations of Serum Sclerostin and Dickkopf-Related Protein-1 Proteins with Future Cardiovascular Events and Mortality in Haemodialysis Patients: A Prospective Cohort Study. Clin. Kidney J..

[52] Goncalves F.L., Elias R.M., dos Reis L.M., Graciolli F.G., Zampieri F.G., Oliveira R.B., Jorgetti V., Moyses R.M. (2014). Serum Sclerostin Is an Independent Predictor of Mortality in Hemodialysis Patients. BMC Nephrol..

[53] Gong L., Zheng D., Yuan J., Cao L., Ni Z., Fang W. (2018). Elevated Levels of Serum Sclerostin Are Linked to Adverse Cardiovascular Outcomes in Peritoneal Dialysis Patients. Int. Urol. Nephrol..

[54] Viaene L., Behets G.J., Claes K., Meijers B., Blocki F., Brandenburg V., Evenepoel P., D’Haese P.C. (2013). Sclerostin: Another Bone-Related Protein Related to All-Cause Mortality in Haemodialysis?. Nephrol. Dial. Transplant..

[55] Kanbay M., Siriopol D., Saglam M., Kurt Y.G., Gok M., Cetinkaya H., Karaman M., Unal H.U., Oguz Y., Sari S. (2014). Serum Sclerostin and Adverse Outcomes in Nondialyzed Chronic Kidney Disease Patients. J. Clin. Endocrinol. Metab..

[56] Drechsler C., Evenepoel P., Vervloet M.G., Wanner C., Ketteler M., Marx N., Floege J., Dekker F.W., Brandenburg V.M., Necosad Study Group (2015). High Levels of Circulating Sclerostin Are Associated with Better Cardiovascular Survival in Incident Dialysis Patients: Results from the Necosad Study. Nephrol. Dial. Transplant..

[57] Jean G., Chazot C., Bresson E., Zaoui E., Cavalier E. (2016). High Serum Sclerostin Levels Are Associated with a Better Outcome in Haemodialysis Patients. Nephron.

[58] Lips L., van Zuijdewijn C.L.M.d., Wee P.M.T., Bots M.L., Blankestijn P.J., van den Dorpel M.A., Fouque D., de Jongh R., Pelletier S., Vervloet M.G. (2017). Serum Sclerostin: Relation with Mortality and Impact of Hemodiafiltration. Nephrol. Dial. Transplant..

[59] Sato M., Hanafusa N., Kawaguchi H., Tsuchiya K., Nitta K. (2018). A Prospective Cohort Study Showing No Association between Serum Sclerostin Level and Mortality in Maintenance Hemodialysis Patients. Kidney Blood Press. Res..

[60] Ge Y., Wu B., Yu X., Wang N., Xu X., Zeng M., Zhang B., Mao H., Xing C. (2021). Association of Serum Sclerostin Level, Coronary Artery Calcification, and Patient Outcomes in Maintenance Dialysis Patients. Blood Purif..

[61] Jorgensen H.S., Winther S., Dupont L., Bottcher M., Rejnmark L., Hauge E.M., Svensson M., Ivarsen P. (2018). Sclerostin Is Not Associated with Cardiovascular Event or Fracture in Kidney Transplantation Candidates. Clin. Nephrol..

[62] Kirkpantur A., Balci M., Turkvatan A., Afsar B. (2016). Serum Sclerostin Levels, Arteriovenous Fistula Calcification and 2-Years All-Cause Mortality in Prevalent Hemodialysis Patients. Nefrologia.

[63] Ishimura E., Okuno S., Ichii M., Norimine K., Yamakawa T., Shoji S., Nishizawa Y., Inaba M. (2014). Relationship between Serum Sclerostin, Bone Metabolism Markers, and Bone Mineral Density in Maintenance Hemodialysis Patients. J. Clin. Endocrinol. Metab..

[64] Kuo T.H., Lin W.H., Chao J.Y., Wu A.B., Tseng C.C., Chang Y.T., Liou H.H., Wang M.C. (2019). Serum Sclerostin Levels Are Positively Related to Bone Mineral Density in Peritoneal Dialysis Patients: A Cross-Sectional Study. BMC Nephrol..

[65] Ho T.Y., Chen N.C., Hsu C.Y., Huang C.W., Lee P.T., Chou K.J., Fang H.C., Chen C.L. (2019). Evaluation of the Association of Wnt Signaling with Coronary Artery Calcification in Patients on Dialysis with Severe Secondary Hyperparathyroidism. BMC Nephrol..

[66] Elsalam M.A., El-Abden M.Z., Mahmoud E., Zahab Z.A., Ahmed H. (2019). Correlation between Serum Sclerostin Level and Bone Density Status in Children on Regular Hemodialysis. Saudi J. Kidney Dis. Transpl..

[67] Szulc P., Boutroy S., Vilayphiou N., Schoppet M., Rauner M., Chapurlat R., Hamann C., Hofbauer L.C. (2013). Correlates of Bone Microarchitectural Parameters and Serum Sclerostin Levels in Men—The Strambo Study. J. Bone Miner. Res..

[68] Atteritano M., Di Mauro E., Canale V., Bruzzese A.M., Ricciardi C.A., Cernaro V., Lacquaniti A., Buemi M., Santoro D. (2017). Higher Serum Sclerostin Levels and Insufficiency of Vitamin D Are Strongly Associated with Vertebral Fractures in Hemodialysis Patients: A Case Control Study. Osteoporos. Int..

[69] Malluche H.H., Davenport D.L., Cantor T., Monier-Faugere M.C. (2014). Bone Mineral Density and Serum Biochemical Predictors of Bone Loss in Patients with Ckd on Dialysis. Clin. J. Am. Soc. Nephrol..

[70] Malluche H.H., Monier-Faugere M.C., Blomquist G., Davenport D.L. (2018). Two-Year Cortical and Trabecular Bone Loss in Ckd-5d: Biochemical and Clinical Predictors. Osteoporos. Int..

[71] Cosman F., Crittenden D.B., Adachi J.D., Binkley N., Czerwinski E., Ferrari S.L., Hofbauer L.C., Lau E., Lewiecki E.M., Miyauchi A. (2016). Romosozumab Treatment in Postmenopausal Women with Osteoporosis. N. Engl. J. Med..

[72] Saag K.G., Petersen J., Brandi M.L., Karaplis A.C., Lorentzon M., Thomas T., Maddox J., Fan M., Meisner P.D., Grauer A. (2017). Romosozumab or Alendronate for Fracture Prevention in Women with Osteoporosis. N. Engl. J. Med..

[73] Lewiecki E.M., Blicharski T., Goemaere S., Lippuner K., Meisner P.D., Miller P.D., Miyauchi A., Maddox J., Chen L., Horlait S. (2018). A Phase Iii Randomized Placebo-Controlled Trial to Evaluate Efficacy and Safety of Romosozumab in Men with Osteoporosis. J. Clin. Endocrinol. Metab..

[74] Lv F., Cai X., Yang W., Gao L., Chen L., Wu J., Ji L. (2020). Denosumab or Romosozumab Therapy and Risk of Cardiovascular Events in Patients with Primary Osteoporosis: Systematic Review and Meta-Analysis. Bone.

[75] Li L., Gong M., Bao D., Sun J., Xiang Z. (2020). Denosumab and Romosozumab Do Not Increase the Risk of Cardiovascular Events in Patients with Primary Osteoporosis: A Reanalysis of the Meta-Analysis. Bone.

[76] Cummings S.R., McCulloch C. (2020). Explanations for the Difference in Rates of Cardiovascular Events in a Trial of Alendronate and Romosozumab. Osteoporos. Int..

[77] Langdahl B.L., Hofbauer L.C., Forfar J.C. (2021). Cardiovascular Safety and Sclerostin Inhibition. J. Clin. Endocrinol. Metab..

[78] Kang J.H., Keller J.J., Lin H.C. (2013). Bisphosphonates Reduced the Risk of Acute Myocardial Infarction: A 2-Year Follow-up Study. Osteoporos. Int..

[79] Kim D.H., Rogers J.R., Fulchino L.A., Kim C.A., Solomon D.H., Kim S.C. (2015). Bisphosphonates and Risk of Cardiovascular Events: A Meta-Analysis. PLoS ONE.

[80] Kranenburg G., Bartstra J.W., Weijmans M., de Jong P.A., Mali W.P., Verhaar H.J., Visseren F.L.J., Spiering W. (2016). Bisphosphonates for Cardiovascular Risk Reduction: A Systematic Review and Meta-Analysis. Atherosclerosis.

[81] Cummings S.R., Lui L.Y., Eastell R., Allen I.E. (2019). Association between Drug Treatments for Patients with Osteoporosis and Overall Mortality Rates: A Meta-Analysis. JAMA Intern. Med..

[82] Vestergaard Kvist A., Faruque J., Vallejo-Yague E., Weiler S., Winter E.M., Burden A.M. (2021). Cardiovascular Safety Profile of Romosozumab: A Pharmacovigilance Analysis of the Us Food and Drug Administration Adverse Event Reporting System (Faers). J. Clin. Med..

[83] FDA (2019). January 16, 2019: Meeting of the Bone, Reproductive and Urologic Drugs Advisory Committee Meeting Announcement.

[84] Cejka D., Parada-Rodriguez D., Pichler S., Marculescu R., Kramer I., Kneissel M., Gross T., Reisinger A., Pahr D., Monier-Faugere M.C. (2016). Only Minor Differences in Renal Osteodystrophy Features between Wild-Type and Sclerostin Knockout Mice with Chronic Kidney Disease. Kidney Int..

[85] Moe S.M., Chen N.X., Newman C.L., Organ J.M., Kneissel M., Kramer I., Gattone V.H., Allen M.R. (2014). Anti-Sclerostin Antibody Treatment in a Rat Model of Progressive Renal Osteodystrophy. J. Bone Miner. Res..

[86] Miller P., Adachi J., Albergari B., Cheung A.M., Chines A., Gielen E., Langdahl B., Miyauchi A., Oates M., Reid I. (2020). Efficacy and Safety of Romosozumab among Postmenopausal Women with Osteoporosis and Mild-to-Moderate Chronic Kidney Disease. Ann. Rheum. Dis..

[87] ClinicalTrials Study of Romosozumab (Amg 785) Administered to Healthy Participants and Patients with Stage 4 Renal Impairment or Stage 5 Renal Impairment Requiring Hemodialysis. https://www.clinicaltrials.gov/ct2/show/results/NCT01833754?term=romosozumab&cond=kidney&draw=2&rank=1.

[88] Amgentrials A Phase 1, Open-Label, Single-Dose Study of Romosozumab (Amg 785) Administered Subcutaneously to Healthy Subjects and Subjects with Stage 4 Renal Impairment or Stage 5 Renal Impairment Requiring Hemodialysis. https://www.amgentrials.com/study/?id=20110227.

[89] Ferrari S.L. (2018). Romosozumab to Rebuild the Foundations of Bone Strength. Nat. Rev. Rheumatol..

[90] Block G.A., Bone H.G., Fang L., Lee E., Padhi D. (2012). A Single-Dose Study of Denosumab in Patients with Various Degrees of Renal Impairment. J. Bone Miner. Res..

[91] Sato M., Inaba M., Yamada S., Emoto M., Ohno Y., Tsujimoto Y. (2021). Efficacy of Romosozumab in Patients with Osteoporosis on Maintenance Hemodialysis in Japan; an Observational Study. J. Bone Miner. Metab..

